# Bereavement interventions to support informal caregivers in the intensive care unit: a systematic review

**DOI:** 10.1186/s12904-021-00763-w

**Published:** 2021-05-12

**Authors:** Stephana J. Moss, Krista Wollny, Therese G. Poulin, Deborah J. Cook, Henry T. Stelfox, Amanda Roze des Ordons, Kirsten M. Fiest

**Affiliations:** 1 Department of Community Health Sciences, Cumming School of Medicine, University of Calgary, Calgary, AB Canada; 2 Department of Critical Care Medicine, University of Calgary, Calgary, AB Canada; 3 O’Brien Institute for Public Health, University of Calgary, Calgary, AB Canada; 4 Hotchkiss Brain Institute, University of Calgary, Calgary, AB Canada; 5 Alberta Health Services, Calgary, AB Canada; 6 Faculty of Nursing, University of Calgary, Calgary, AB Canada; 7 Alberta Children’s Hospital Research Institute, Calgary, AB Canada; 8 Department of Medicine, McMaster University, Hamilton, ON Canada; 9 Department of Health Research Methods, Evidence, and Impact, McMaster University, Hamilton, ON Canada; 10 Division of Palliative Medicine, Department of Oncology, Cumming School of Medicine, Calgary, AB Canada; 11 Department of Psychiatry, Cumming School of Medicine, University of Calgary, Calgary, AB Canada

**Keywords:** Bereavement, Interventions, Intensive care unit, Critical care, Informal caregiver, Coping

## Abstract

**Background:**

Informal caregivers of critically ill patients in intensive care unit (ICUs) experience negative psychological sequelae that worsen after death. We synthesized outcomes reported from ICU bereavement interventions intended to improve informal caregivers’ ability to cope with grief.

**Data sources:**

MEDLINE, EMBASE, CINAHL and PsycINFO from inception to October 2020.

**Study selection:**

Randomized controlled trials (RCTs) of bereavement interventions to support informal caregivers of adult patients who died in ICU.

**Data extraction:**

Two reviewers independently extracted data in duplicate. Narrative synthesis was conducted.

**Data synthesis:**

Bereavement interventions were categorized according to the UK National Institute for Health and Clinical Excellence three-tiered model of bereavement support according to the level of need: (1) *Universal information* provided to all those bereaved; (2) *Selected or targeted non-specialist support* provided to those who are at-risk of developing complex needs; and/or (3) *Professional specialist interventions* provided to those with a high level of complex needs. Outcome measures were synthesized according to core outcomes established for evaluating bereavement support for adults who have lost other adults to illness.

**Results:**

Three studies of ICU bereavement interventions from 31 ICUs across 26 hospitals were included. One trial examining the effect of family presence at brain death assessment integrated all three categories of support but did not report significant improvement in emotional or psychological distress. Two other trials assessed a condolence letter intervention, which did not decrease grief symptoms and may have increased symptoms of depression and post-traumatic stress disorder, and a storytelling intervention that found no significant improvements in anxiety, depression, post-traumatic stress, or complicated grief. Four of nine core bereavement outcomes were not assessed anytime in follow-up.

**Conclusions:**

Currently available trial evidence is sparse and does not support the use of bereavement interventions for informal caregivers of critically ill patients who die in the ICU.

**Supplementary Information:**

The online version contains supplementary material available at 10.1186/s12904-021-00763-w.

## Introduction

Informal caregivers (i.e., family, friends) of critically ill patients in the intensive care unit (ICU) experience negative psychological and emotional sequelae [1, 2] that worsen after patient death [3, 4]. Despite how common death is among patients admitted to the ICU [5, 6], preparing informal caregivers to cope with their grief is challenging [7]. Withholding and withdrawing life-sustaining treatment while mitigating suffering in the ICU is extremely complex [8]. Several groups have taken a leading role in developing national, cultural-specific guidelines and recommendations for healthcare professional to support the bereavement process in daily practice within different ethical environments [9–11].

A 2019 narrative review reported inconsistent evidence for the association between bereavement support in adult ICU and informal caregiver outcomes, noting methodological shortcomings in the evidence [12]. Since 2019, a set of core outcomes was developed to address inconsistent evaluation of bereavement services and models of support for informal caregivers in adult palliative care [13]. The scope of the core outcomes set (e.g., ability to cope with grief, quality of life and mental well-being) is for bereavement research and clinical practice generally, and was designed to assess bereavement interventions for adults whose adult friends and family members have died. The core outcomes set is comprised of 21 caregiver-level outcomes representing nine categories (e.g., negative and overwhelming grief, communication and connectedness) within two primary domains (i.e., ability to cope with grief; quality of life and mental well-being). The core outcomes set developed for adult palliative care settings [13] is relevant for use in ICU given that death in ICU is common and that bereavement interventions to prepare informal caregivers to cope with their grief may be appropriate across the entire critical illness trajectory [14].

The aims of this review were to map bereavement interventions to established core outcomes for evaluating bereavement support among informal caregivers, and to identify grief support interventions that improve informal caregivers’ ability to cope with the grief.

## Methods

This protocol-based systematic review (PROSPERO ID: CRDCRD42020202908) was reported in accordance with the Preferred Reporting Items for Systematic Review and Meta-analyses (PRISMA) guideline [15] (Supplemental Table 1).

### Identification and selection of studies

We searched MEDLINE, EMBASE, CINAHL and PsycINFO from inception to October 01, 2020. A medical librarian (D.L.L.) assisted with the development, piloting, and execution of searches (Supplemental Table 2). No language or date restrictions were applied. Reference lists of included papers were reviewed to identify potentially missed studies.

### Study eligibility

Two reviewers (S.J.M. and T.G.P.) independently evaluated all records for eligibility in two stages. In the title and abstract stage, any record selected by either reviewer as meeting one (or more) eligibility criteria progressed to the full-text review stage. Studies were eligible for inclusion if both reviewers agreed that the study met all eligibility criteria following review of the full-text. Disagreements were resolved through consensus with another author (K.W.).

We included quantitative, experimental studies reporting randomized controlled trials of bereavement interventions to support informal caregivers of adult patients who died in ICU. We excluded interventions for health care professionals and interventions that were conducted prior to patient death (i.e., at end-of-life). We included studies where the intervention was performed outside ICU (e.g., home follow-up). For the purposes of our review, we defined ICU bereavement interventions as services healthcare professionals provide or coordinate for informal caregivers of critically ill patients after patient death (including brain death) in the ICU [16, 17]. We defined an informal caregiver as any informal (i.e., non-clinical) person who regularly provides patient support and is in some way implicated in patient care or directly affected by patient health (e.g., family, friend) [18], and critically ill patients as any persons currently or previously admitted to ICU [18]. In addition to studies that reported on patient death, we included studies where brain death was considered as patient death [19], since previous work indicates informal caregivers accept brain death as patient death [20] and caregiver grieving processes are similar [21]. The trials investigated bereavement interventions for informal caregivers of adult patients (> 17 years) that applied at least one (or more) support category from the UK National Institute for Health and Clinical Excellence [22, 23] three-tiered model of bereavement support, that includes: (1) *Universal information* provided to all bereaved; (2) *Selected or targeted non-specialist support* provided to those with a medium level of need who are at-risk of developing complex needs; and/or (3) *Indicated professional specialist interventions* provided to those with a high level of complex needs. Any one bereavement intervention could have applied multiple categories (i.e., types) of support, but needed to report at least category to be included in our review. Finally, to be eligible for inclusion, we required that the interventions reported on at least one of 21 caregiver-level outcomes within at least one of nine categories from the core set of outcomes for evaluating bereavement support for adult caregivers in adult care settings [13] (Supplemental Table 3). References were managed in Endnote X9 (Clarivate Analytics, Philadelphia, PA, USA).

### Data extraction and risk of Bias assessment

Two reviewers (S.J.M., K.W.) used structured forms developed by the study team to extract information independently and in duplicate for each included study. Information on document characteristics (e.g., year of publication, geographic location), study characteristics (e.g., setting, sites), patient and caregiver characteristics (e.g., age, relationship), intervention characteristics (e.g., type of support and target population level of need, follow-up), core outcomes (e.g., negative mental and emotional state, participation in work and/or other regular activities), statistical significance (e.g., *p*-values, measures of variance), and authors’ conclusions were collected. Risk of bias for objective (i.e., measurement-based) outcomes was independently assessed by two reviewers (S.J.M. and K.W.) using the Cochrane Collaboration’s tool to rate studies at low, high or unclear risk of bias [24]. We (S.J.M. and K.W.) independently assessed quality of core outcomes using the BMJ Best Practice GRADE of Evidence Tool [25].

### Synthesis

A narrative synthesis was conducted for all trials. We mapped primary and secondary outcomes reported in the included studies to the standardized core outcomes set for bereavement interventions [13] to help guide future research in ICU bereavement care [26]. To account for variability in the timing of study end-points, we used common clinically relevant follow-up periods of 1- to 3-month and 4- to 6-month follow-up. To facilitate comparison among the different instruments used to evaluate core outcomes, standardized mean differences (SMDs) with corresponding 95% confidence intervals (CIs) were calculated using a Hedges adjusted *g* estimator to correct for small sample bias [24]. For studies that reported medians or proportions, we contacted the primary author to obtain the corresponding mean and standard deviation (SD). For studies in which authors did not provide additional data, we estimated the mean and SD using validated estimations [27]. If necessary, individual study results were corrected for directionality such that higher coping scores represented better ability to cope, and lower wellbeing scores indicated worse wellbeing. Heterogeneity across a small number of trials precluded pooling SMDs in meta-analysis.

## Results

### Study characteristics

**Fig. 1:**
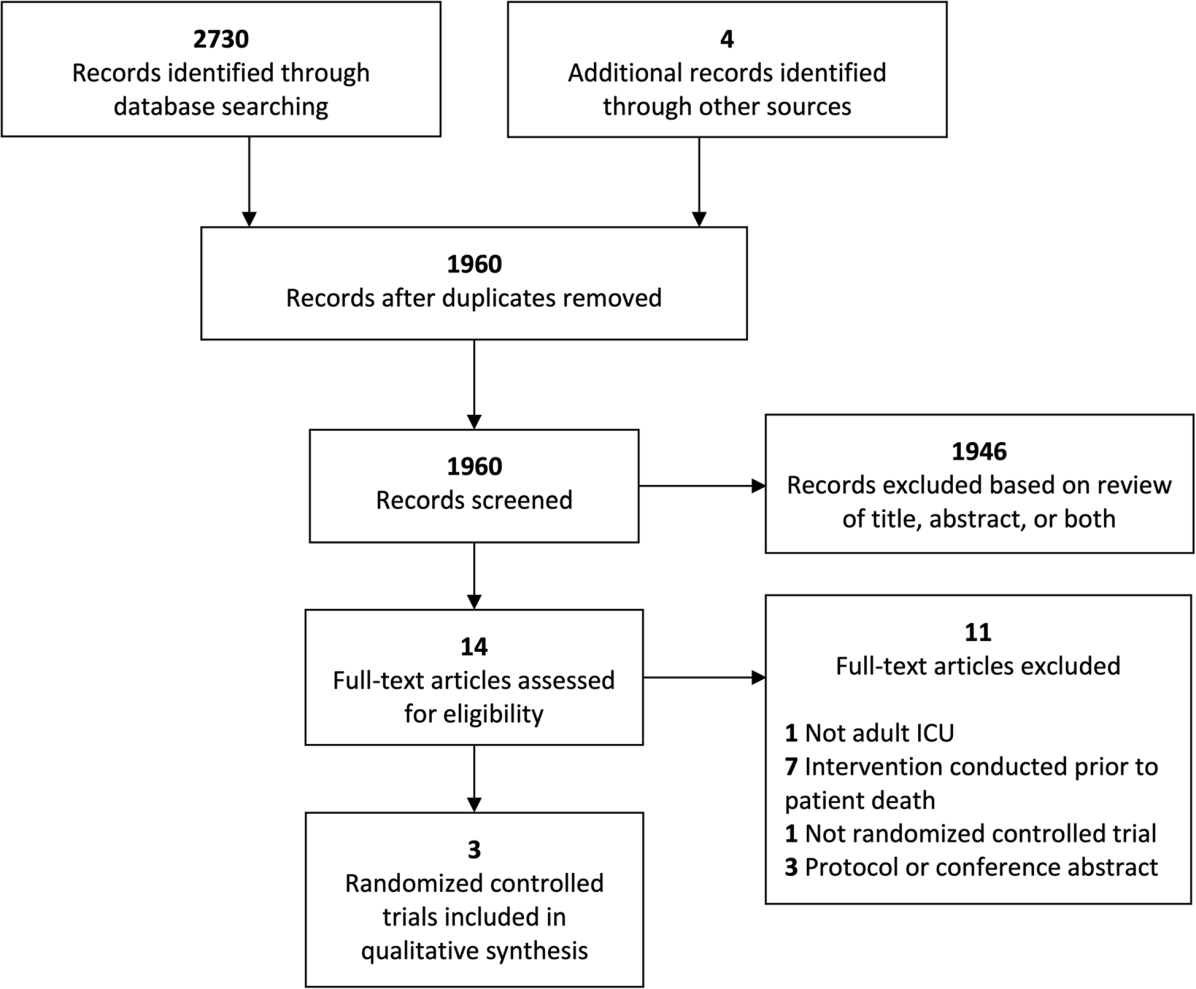
Results of Literature Searches to Identify Randomized Controlled Trials of ICU Bereavement Interventions

Searches identified 1960 unique records, of which 14 were potentially relevant based on initial title and abstract screening (Fig. 1). Following full-text review, three of the 14 articles were included, describing three unique adult ICU bereavement interventions for 296 informal caregivers from 31 ICUs across 22 hospitals in France [28] and 4 in the United States [29, 30] (Table 1). Reasons for exclusion of 11 articles reviewed in full-text are provided in Supplemental Table 3. One trial conducted the ICU bereavement intervention after patient death with a single follow-up time point within 1- to 3-months [30]. The other two trials conducted interventions at 2 weeks following patient death with 1- and 6-month follow-up [28], and at 4 weeks following patient death with 3- and 6-month follow-up [29]. One trial integrated all three categories of support (i.e., universal information, selected or targeted non-specialist support, and an indicated professional specialist intervention) within their bereavement intervention [30]. The other two trials incorporated either selected or targeted non-specialist support with an indicated professional specialist intervention [28], or an indicated professional specialist intervention alone [29]. Included studies assessed in sum four (of nine) core outcomes categories [14 of 21 individual outcomes] that included: (1) negative and overwhelming grief; (3) understanding, accepting and finding meaning in grief; and (3) accessing appropriate support [relating to caregiver ability to cope with grief] (Supplemental Tables 5 and 6), as well as (4) participation in work and/or other regular activities and (5) negative mental and emotional state [relating to caregiver quality of life and mental wellbeing] (Supplemental Tables 7 and 8). All trials were judged as having low risk of bias (Table 2). Overall, the quantity of evidence on bereavement interventions for informal caregivers in adult ICUs is low (Table 3).

**Table 1:** Study Characteristics and Patient and Informal Caregiver Demographics from Randomized Controlled Trials of ICU Bereavement Interventions

Source	Hospital and ICU Settings and Types	Bereavement Intervention	Duration of Intervention	Follow-Up Timepoints	Patient Demographics *(of those randomized)*	Caregiver Demographics *(of those randomized)*
Barnato et al., 2017 [29]	5 ICUs 1 trauma 1 cardiovascular 2 medical 1 mixed 3 hospitals 1 tertiary teaching 1 cancer center 1 community	Storytelling delivered via home visit or telephone call approximately 4 weeks following patient death that included: - non-judgmental elicitation of the story of the events leading up to the patient’s ICU admission - ICU experience and decision process - aftermath of the patient’s death	1–2 h	Follow-up assessments conducted via telephone interview or by mail at: 3 months 6 months	Total *N* = 32 *n, intervention = 18* age, 67.8 yr (SD 13.7) female, 50% *n, control = 14* age, 72.0 yr (SD 10.2) female, 50%	Total *N* = 32 *n, intervention = 18* age, 55.0 yr (SD 11.0) female, 61.1% *n, control = 14* age, 55.9 yr (SD 12.6) female, 86.7%
Kentish-Barnes et al., 2017 [28]	22 ICUs 8 medical 11 general 1 surgical 1 nephrology 1 anesthesia-surgical 22 hospitals 11 academic 11 non-academic	Condolence letter prepared (hand-written) within 3 days after patient death and sent by standard mail 15 days after patient death that included: - recognition of the death - name of the decreased - mention of a personal impression - recognition of the family member - offer to help - express sympathy	Not reported	Follow-up assessments conducted via telephone interview by psychologists, sociologists, and research nurses blinded to study group at: 1 month 6 months	Total *N* = 242 *n, intervention = 123* age, 61 yr (Rg. 54–71) female, 33.3% *n, control = 119* age, 61 yr (Rg. 54–66) female, 37.0%	Total *N* = 242 *n, intervention = 123* age, 57 yr (Rg. 46–65.5) female, 67.9% *n, control = 119* age, 56 yr (Rg. 44–64.5) female, 71.7%
Tawil et al., 2014 [30]	4 ICUs 1 medical 1 neurosciences 1 trauma/surgical 1 PICU (> 17 yr eligible) 1 hospital 1 tertiary teaching	Family groups presence during brain death evaluation joined the evaluating physician at the patient’s bedside to observe the brain death evaluation including all brainstem reflex testing and the apnea test. The subjects were accompanied by a chaperone who could explain the process and answer questions during the evaluation. After the brain death evaluation was complete, the family members were notified of the results and given an opportunity to ask questions.	Average duration of brain death evaluation not stated	All family members sent hard copies of assessment surveys then telephoned by a trained research nurse who administered the surveys and recorded responses within 1- to 3-months after patient death	Total *N* = 17 *n, intervention = 11* age, 41.7 yr (Rg. 19–67) female, 55% *n, control = 6* age, 52.5 yr (Rg. 32–67) female, 33%	Total *N* = 58 *n, intervention = 38* age, 41.7 yr (SD 14.4) female, 61.1% *n, control = 20* age, 44.6 yr (SD 17.6) female, 85.7%

*ICU* intensive care unit; *RG* range; *SD* standard deviation; *YR* year

**Table 2:** Risk of Bias Among Randomized Controlled Trials of ICU Bereavement Intervention^1^

Study	Random sequence generation	Allocation concealment	Blinding of participants, researchers	Blinding of outcome assessment	Incomplete outcome data^**2**^	Selective reporting
Barnato et al., 2017 [29]	Low	Low	High	Low	High	Low
Kentish-Barnes et al., 2017 [28]	Low	Low	High	Low	High	Low
Tawil et al., 2014 [30]	Low	Low	High	Unclear	High	Low

^1^Determined by the Cochrane Risk of Bias Assessment Tool

^2^Overall attrition above 20% represents high risk of attrition bias; attrition below 20% and unequal between intervention and control group represents high risk of attrition bias; ratings of unclear represent that either overall attrition or attrition between groups was not reported

**Table 3:** Summary of Findings Among Randomized Controlled Trials of ICU Bereavement Interventions

Outcome	Assessed By	Follow-Up Range	No. Studies	No. Caregivers^**1**^	GRADE of Evidence^**2**^
*Ability to Cope with Grief*					
Negative and overwhelming grief	Decision Regret Scale	6-month	1	30	Low
Communication and connectedness	None	N/A	0	0	N/A
Understanding, accepting and finding meaning in grief	Inventory of Complicated Grief	1-month to 6-month	2	220	Moderate
Finding balance between grief and life going forwards	None	N/A	0	0	N/A
Accessing appropriate support	Single Yes/No Question	3-month to 6-month	1	30	Low
*Quality of Life and Metal Wellbeing*					
Participation in work and/or other regular activities	General Health Questionnaire-12	1-month to 3-months	1	58	Low
Relationships and social functioning	None	N/A	0	0	N/A
Positive mental wellbeing	None	N/A	0	0	N/A
Negative mental and emotional state	Impact of Event Scale; Impact of Event Scale-Revised; Hospital Anxiety and Depression-Anxiety; Hospital Anxiety and Depression-Depression; Hospital Anxiety and Depression-Total; Patient Health Questionnaire-9; Post-Intensive Care Syndrome-Family	1-month to 6-month	3	278	Moderate

*N/A* not applicable

^1^At last timepoint of follow-up

^2^Determined by the BMJ Best Practice GRADE of Evidence Assessment Tool; reasons for downgrade related mainly to lack of evidence

### Three-tiered model of bereavement support and assessment of Core outcomes

Table 2 provides a summary of findings among RCTs of ICU bereavement interventions whilst SDMs with corresponding interpretation of effect of intervention are in Supplemental Tables 5, 6, 7, and 8. Among the three included trials, no significant effect of intervention was determined for negative and overwhelming grief (assessed by the Decision Regret Scale in one study); accessing appropriate support (assessed by yes/no questions in two studies); and participation in work and/or other regular activities (assessed using the General Health Questionnaire-12 in one study). Mixed results (both negative and non-significant) were determined for understanding, accepting and finding meaning in grief (assessed by the Inventory of Complicated Grief in two studies) and negative mental and emotional state (assessed by the Impact of Event Scale; Impact of Event Scale-Revised; Hospital Anxiety and Depression-Anxiety; Hospital Anxiety and Depression-Depression; Hospital Anxiety and Depression-Total; Patient Health Questionnaire-9; and Prevalence of Post-Intensive Care Syndrome-Family in all three studies).

Two trials integrated multiple categories of bereavement support [28, 30] proposed by the UK National Institute for Health and Clinical Excellence [22] to be made available according to the level of need. Effect of family presence compared to absence at brain death evaluation investigated by Tawil et al. [30] across four ICUs within a single academic hospital, incorporated universal information [for all levels of need], selected or targeted non-specialist support [for medium level of need], and an indicated professional specialist intervention [for high and complex level of need] for 38 informal caregivers of adult patients in ICU whom the treating intensivist suspected had suffered brain death. Multiple caregivers per patient joined the evaluating physician at the patient’s bedside to observe the brain death evaluation. Caregivers were accompanied by a chaperone who explained the process and were available to answer questions during the evaluation. Caregivers randomized to be absent during the evaluation waited in an adjacent room, accompanied by a chaperone. After the brain death evaluation was complete, informal caregivers were notified of the results and given an opportunity to ask questions. All informal caregivers were sent infographics and hard copies of assessment surveys, then telephoned by a trained research nurse who administered the surveys and recorded responses within 1- to 3-months after patient death. Investigators found no significant improvement in emotional or psychological distress, or participation in work and/or other regular activities (e.g., daily tasks, social activities) up to 3-months. Investigators concluded that informal caregiver presence during brain death evaluation is feasible and safe.

Kentish-Barnes et al. [28] conducted a randomized parallel-group trial across 22 ICUs within 22 hospitals (11 academic and 11 non-academic). They facilitated two categories of bereavement support (i.e., selected or targeted non-specialist support [for medium level of need] with an indicated professional specialist intervention [for high and complex level of need]) to contribute evidence on two core outcomes (i.e., understanding, accepting and finding meaning in grief and negative mental and emotional state). They prepared a hand-written condolence letter for 123 informal caregivers sent within 15 days after patient death that included: (1) recognition of the death; (2) name of the deceased; (3) mention of a personal impression; (4) recognition of the informal caregiver; (5) offer to help; and (6) expression of sympathy. Their 1- and 6-month telephone follow-up assessments were conducted by any one of a psychologist, sociologist or research nurse. Condolence letters had no effect on any core outcome at 1-month follow-up. At 6-month follow-up they reported that among informal caregivers of patients who died in ICU, a condolence letter failed to alleviate grief symptoms and increased depression and and post-traumatic stress disorder-related symptoms.

A pilot single-blind trial across five ICUs within three hospitals (one academic) incorporated one category of bereavement support providing one professional specialist intervention for a high and complex level of need [29]. The storytelling intervention delivered by Barnato et al. [29] to 18 caregivers via home visit or telephone approximately 4 weeks following patient death consisted of: (1) non-judgmental elicitation of the story of the events leading up to the patient’s ICU admission; (2) description of the ICU experience and decision process; and (3) summary of the aftermath of the patient’s death. Follow-up was performed at 3- and 6-months, with rationale to assess selection bias rather than to provide additional opportunity for support. The authors reported that their storytelling intervention met all a priori feasibility, tolerability and acceptability targets and there were no significant improvements in anxiety, depression, post-traumatic stress or complicated grief at any time-point of follow-up. The authors noted that their sample size was too small to make any inferences about the effect of the storytelling intervention on individual psychological symptoms.

### Core outcomes not assessed

No trial reported on the effect of an ICU bereavement intervention on four of nine core outcomes categories at any time of follow-up that included: (1) communication and connectedness; (3) finding balance between grief and life going forwards; and (4) relationships and social functioning; and (5) positive mental wellbeing.

## Discussion

This systematic review and narrative synthesis of bereavement interventions for informal caregivers of adult patients who died in ICU identified family presence during brain death evaluation and storytelling were feasible and acceptable among caregivers, although none were found to improve their emotional and psychological wellbeing up to 3-month and 6-months in follow up, respectively. Condolence letters provided to caregivers may worsen depression and post-traumatic stress disorder-related symptoms at 6-month follow-up. No included bereavement intervention alleviated grief symptoms or improved ability to cope.

Overall, despite the low risk of bias of these trials, the body of evidence on bereavement interventions for informal caregivers in adult ICUs is too modest to know whether bereavement interventions initiated following ICU patient death adequately prepare caregivers to cope with their grief. Other relevant outcomes that could be incorporated in future interventions is underscored by how four of nine categories from the core outcomes set for evaluating bereavement interventions remain to be investigated in RCTs in this field, including (1) communication and connectedness; (2) finding balance between grief and life going forwards; and (3) relationships and social functioning; and (4) positive mental wellbeing.

This systematic review adds to the literature by: (1) categorizing bereavement interventions according to the three-tiered model of bereavement support from the UK National Institute for Health and Clinical Excellence [22] and (2) mapping available evidence to a core set of standardized outcomes reported from bereavement interventions in adult ICUs [13]. Our aggregate, narrative synthesis on categories of bereavement support and assessment of core outcomes offers considerations for future trials on ICU bereavement interventions [26]. Our results should be interpreted cautiously given the dearth of research on this topic, underscoring the need for further studies to develop and evaluate effective bereavement interventions in adult ICU.

Though the bereavement interventions we reviewed were generally appreciated by informal caregivers, all authors noted that what constitutes appropriate bereavement care and adequate follow-up is unclear. Clinicians are uncertain how to provide ICU bereavement interventions to manifest support rather than to reduce grief symptom outcomes [28]. The phenomenon of worsened psychological outcomes after mental health interventions has previously been reported in relation to psychological debriefing for preventing post-traumatic stress disorder [31]. Individuals cope differently with loss, such that any intervention that changes coping trajectories has the potential to do harm [32]. The available evidence suggests that clinicians should consider adopting the approach from bereavement therapy [33] that considers grief as a natural process with a variety of healthy responses to loss. It is possible that there are many effective ways to support bereaved informal caregivers in the ICU.

### Strengths and weaknesses

Though we used a broad definition to identify trials that employed bereavement interventions consistent with any one of the support categories from the three-tiered model of bereavement support, [22] bereavement interventions in adult ICU have been evaluated by few studies. In the absence of more extensive global research on this topic, findings on bereavement interventions need to be considered within the context of the individual studies in which they were performed. For example, in France, physicians have final authority regarding decisions to forgo life-sustaining treatments [2, 34], meaning that effectiveness of bereavement interventions may vary according to sociocultural circumstances, including the degree of family involvement and sense of responsibility in shared decision making [35, 36].

This review has several limitations and our results should be interpreted cautiously. First, we excluded quasi-experimental, observational and qualitative studies, many of which have suggested or demonstrated benefits of bereavement care and provided insights from different perspectives [37–39]. Second, this review did not include conventional palliative care interventions (neither early nor at end-of-life) despite prevailing views that palliative care interventions are appropriate throughout the critical illness trajectory [14]. Third, quantitative results from these studies should be interpreted cautiously given that they were underpowered; furthermore, the design of these three RCTs precluded a quantitative meta-analysis. Fourth, we used the Cochrane Risk of Bias tool to perform risk of bias assessments, which is not designed to consider multi-component behavior change interventions [24]. Fifth, though we did not employ language or date restrictions, our review is restricted to countries represented by the included studies and should not be taken to represent a generalizable, global report on the state of bereavement interventions for adult ICU caregivers. Sixth, we did not include preliminary findings from conference proceedings or planned interventions from protocols [40, 41].

### Unanswered questions and future research

Bereavement interventions in adult ICU remains an active area of research with many gaps in our understanding. Given the complexities and inconsistencies in limited bereavement interventions, we were unable to determine effective components of bereavement interventions and how specific categories of support targeted to the caregivers’ level of need might affect their ability to cope. Single-component bereavement interventions offer a potentially resource-efficient means of preparing informal caregivers to cope with grief and sustain mental wellbeing following death of a loved one in ICU [42, 43]. However, it is possible that multi-component bereavement interventions bundles might be more effective [44].

From an intervention perspective, future research is needed to identify efficacious components of bereavement care. Informal caregivers may require different bereavement intervention components at different timepoints along the critically illness trajectory. Future trials should consider active comparisons of different components of ICU bereavement care strategies. From an outcomes perspective, to consolidate robust evidence corresponding to core outcomes, future studies should use standardized and validated measures appropriate for informal caregivers at clinically relevant follow-up time points. Four of nine categories from the core outcomes set for evaluating bereavement interventions in palliative care remain to be investigated in adult ICUs, including three coping-related outcomes and two mental wellbeing outcomes. From a process evaluation perspective, future studies should describe intervention fidelity, dose, and reach, to ensure interventions are consistently provided as intended and reliably adhered to as required for optimized impact of bereavement care.

## Conclusions

In our systematic review and narrative synthesis of RCTs evaluating bereavement interventions in adult ICUs targeting for informal caregivers, we found that the evidence is modest in scope. Deeper understanding of what interventions are most effective, for whom, at what time, and in which contexts, is required. Based on currently available trial data, there are not any specific bereavement interventions that can be firmly recommended to help prepare caregivers of critically ill patients to cope with their grief.

## Supplementary Information

**Additional file 1.**

## Data Availability

Data sharing is not applicable to this article as no datasets were generated or analyzed during the current study.
