# Supplementary material for: Bereavement interventions to support informal caregivers in the intensive care unit: a systematic review
Source: BMC Palliat Care. 2021 May 12;20:66. doi: 10.1186/s12904-021-00763-w (PMC8117265; doi:10.1186/s12904-021-00763-w)
Supplement: Supplementary file 1 — Additional file 1. [file 12904_2021_763_MOESM1_ESM.docx]

**Bereavement Interventions to Support Informal Caregivers in the Intensive Care Unit: A Systematic Review**

*Supplemental Information*

Stephana J. Cherak, MSc PhD Candidate^1,2,3,4^; Krista Wollny, RN MN PhD Candidate^1,5,6,7^; Therese G. Poulin, BSc Student^1^; Deborah J. Cook, MD MSc^9,10^; Henry T. Stelfox, MD PhD^1,2,3,5^; Amanda Roze des Ordons, MD MMEd^2,5,11^*; Kirsten M. Fiest, PhD^1,2,3,4,5,8^*

***indicates co-senior authors**

^1^Department of Community Health Sciences, Cumming School of Medicine, University of Calgary, Calgary, AB, Canada

^2^Department of Critical Care Medicine, University of Calgary, Calgary, AB, Canada

^3^O’Brien Institute for Public Health, University of Calgary, Calgary, AB, Canada

^4^Hotchkiss Brain Institute, University of Calgary, Calgary, AB, Canada

^5^Alberta Health Services, Calgary, AB, Canada

^6^Faculty of Nursing, University of Calgary, Calgary, AB, Canada

^7^Alberta Children’s Hospital Research Institute, Calgary, AB, Canada

^8^Department of Psychiatry, Cumming School of Medicine, University of Calgary, Calgary AB, Canada

^9^Department of Medicine, McMaster University, Hamilton ON, Canada

^10^Department of Health Research Methods, Evidence, and Impact, McMaster University, Hamilton ON, Canada

^11^Division of Palliative Medicine, Department of Oncology

**Supplemental Table 1.** Preferred Reporting Items for Systematic Reviews and Meta-Analyses (PRISMA) Checklist

| **Section/topic** | **#** | **Checklist item** | **Reported on page #** |
| --- | --- | --- | --- |
| **TITLE** | | |  |
| Title | 1 | Identify the report as a systematic review, meta-analysis, or both. | 1 |
| **ABSTRACT** | | |  |
| Structured summary | 2 | Provide a structured summary including, as applicable: background; objectives; data sources; study eligibility criteria, participants, and interventions; study appraisal and synthesis methods; results; limitations; conclusions and implications of key findings; systematic review registration number. | 4-5 |
| **INTRODUCTION** | | |  |
| Rationale | 3 | Describe the rationale for the review in the context of what is already known. | 6 |
| Objectives | 4 | Provide an explicit statement of questions being addressed with reference to participants, interventions, comparisons, outcomes, and study design (PICOS). | 6 |
| **METHODS** | | |  |
| Protocol and registration | 5 | Indicate if a review protocol exists, if and where it can be accessed (e.g., Web address), and, if available, provide registration information including registration number. | 7 |
| Eligibility criteria | 6 | Specify study characteristics (e.g., PICOS, length of follow-up) and report characteristics (e.g., years considered, language, publication status) used as criteria for eligibility, giving rationale. | 7 |
| Information sources | 7 | Describe all information sources (e.g., databases with dates of coverage, contact with study authors to identify additional studies) in the search and date last searched. | 7 |
| Search | 8 | Present full electronic search strategy for at least one database, including any limits used, such that it could be repeated. | 7  S. Table 2 |
| Study selection | 9 | State the process for selecting studies (i.e., screening, eligibility, included in systematic review, and, if applicable, included in the meta-analysis). | 8 |
| Data collection process | 10 | Describe method of data extraction from reports (e.g., piloted forms, independently, in duplicate) and any processes for obtaining and confirming data from investigators. | 8 |
| Data items | 11 | List and define all variables for which data were sought (e.g., PICOS, funding sources) and any assumptions and simplifications made. | 8-9 |
| Risk of bias in individual studies | 12 | Describe methods used for assessing risk of bias of individual studies (including specification of whether this was done at the study or outcome level), and how this information is to be used in any data synthesis. | 8 |
| Summary measures | 13 | State the principal summary measures (e.g., risk ratio, difference in means). | 8-9 |
| Synthesis of results | 14 | Describe the methods of handling data and combining results of studies, if done, including measures of consistency (e.g., I^2^) for each meta-analysis. | 8-9 |

| **Section/topic** | **#** | **Checklist item** | **Reported on page #** |
| --- | --- | --- | --- |
| Risk of bias across studies | 15 | Specify any assessment of risk of bias that may affect the cumulative evidence (e.g., publication bias, selective reporting within studies). | 8 |
| Additional analyses | 16 | Describe methods of additional analyses (e.g., sensitivity or subgroup analyses, meta-regression), if done, indicating which were pre-specified. | 8-9 |
| **RESULTS** | | |  |
| Study selection | 17 | Give numbers of studies screened, assessed for eligibility, and included in the review, with reasons for exclusions at each stage, ideally with a flow diagram. | 9-10 |
| Study characteristics | 18 | For each study, present characteristics for which data were extracted (e.g., study size, PICOS, follow-up period) and provide the citations. | 9-10  Table 1 |
| Risk of bias within studies | 19 | Present data on risk of bias of each study and, if available, any outcome level assessment (see item 12). | S. Table 8 |
| Results of individual studies | 20 | For all outcomes considered (benefits or harms), present, for each study: (a) simple summary data for each intervention group (b) effect estimates and confidence intervals, ideally with a forest plot. | S. Table 4  S. Table 5  S. Table 6  S. Table 7 |
| Synthesis of results | 21 | Present results of each meta-analysis done, including confidence intervals and measures of consistency. | N/A |
| Risk of bias across studies | 22 | Present results of any assessment of risk of bias across studies (see Item 15). | Table 2 |
| Additional analysis | 23 | Give results of additional analyses, if done (e.g., sensitivity or subgroup analyses, meta-regression [see Item 16]). | N/A |
| **DISCUSSION** | | |  |
| Summary of evidence | 24 | Summarize the main findings including the strength of evidence for each main outcome; consider their relevance to key groups (e.g., healthcare providers, users, and policy makers). | 12-13 |
| Limitations | 25 | Discuss limitations at study and outcome level (e.g., risk of bias), and at review-level (e.g., incomplete retrieval of identified research, reporting bias). | 14-15 |
| Conclusions | 26 | Provide a general interpretation of the results in the context of other evidence, and implications for future research. | 16 |
| **FUNDING** | | |  |
| Funding | 27 | Describe sources of funding for the systematic review and other support (e.g., supply of data); role of funders for the systematic review. | 3 |

*From:*  Moher D, Liberati A, Tetzlaff J, Altman DG, The PRISMA Group (2009). Preferred Reporting Items for Systematic Reviews and Meta-Analyses: The PRISMA Statement. PLoS Med 6(7): e1000097. doi:10.1371/journal.pmed1000097

**Supplemental Table 2.** MEDLINE Database Search Strategy

| **Population**  *(Informal or Family Caregivers)* | **Setting**  *(Adult Intensive Care Unit)* | **Interventions**  *(Bereavement)* | **All**  *(Combined)* |
| --- | --- | --- | --- |
| 1. Exp Caregivers/ 2. Exp Family/ 3. Family.mp 4. Families.mp. 5. Caregiv*.mp. 6. “care giv*”.mp. 7. Carer*.mp. 8. Spouse.mp. 9. “next of kin".mp. 10. Support person.mp. 11. “loved one*”.mp. 12. “Significant other*”.mp. 13. Partner.mp. 14. Relative.mp. 15. Proxy.mp. 16. Surrogate.mp. 17. Friend.mp. 18. Or/1-17 | 1. Exp Intensive Care Unit/ 2. “intensive care”.mp. 3. ICU.mp. 4. Critical* ill.mp. 5. “critical care”.mp. 6. Or/19-23 | 1. Exp bereavement/ 2. Exp grief/ 3. (bereave* or grief or griev* or mourn*).tw. 4. (sorrow* or sadness).tw. 5. widowhood/ 6. widow*.tw. 7. Or/25-30 | 1. 18 and 24 and 31 |

mp=title, abstract, original title, name of substance word, subject hearing word, keyword heading word, protocol supplementary concept word, rare disease supplementary concept word, unique identifier

**Supplemental Table 3.** Reasons for Exclusion of Articles Reviewed in Full-Text

| **Excluded Articles that were Reviewed in Full-Text** | | | | |
| --- | --- | --- | --- | --- |
| *Author, Year* | *Adult Intensive Care Unit* | *Intervention Conducted After Patient Death^1^* | *Randomized Controlled Trial* | *Peer-reviewed Publication^2^* |
| Carson, 2016 | ✓ | X | ✓ | ✓ |
| Combe, 2005 | ✓ | ✓ | X | X |
| Cox, 2019 | ✓ | X | ✓ | ✓ |
| Curtis, 2016 | ✓ | X | ✓ | ✓ |
| Kaufer, 2008 | X | ✓ | ✓ | ✓ |
| Kentish-Barnes, 2018 | ✓ | ✓ | ✓ | X |
| Lautrette, 2007 | ✓ | X | ✓ | ✓ |
| Nielson, 2019 | ✓ | X | ✓ | ✓ |
| Prigerson, 2020 | ✓ | ✓ | ✓ | X |
| Rosenbaum, 2015 | ✓ | X | ✓ | ✓ |
| White, 2018 | ✓ | X | ✓ | ✓ |

^1^As opposed to the intervention being conducted at end-of-life (prior to patient death) and with follow-up occurring after patient death

^2^That excludes protocols and conference abstract publications

1. Carson SSC, Christopher E.; Wallenstein, Sylvan; Hanson, Laura C.; Danis, Marion; Tulsky, James A.; Chai, Emily; Nelson, Judith E.: Effect of palliative care-led meetings for families of patients with chronic critical illness: A randomized clinical trial. JAMA: Journal of the American Medical Association 2016; 316(1):51-62
2. Combe D: The use of patient diaries in an intensive care unit. Nursing in critical care 2005; 10(1):31-34
3. Cox CEW, Douglas B.; Hough, Catherine L.; Jones, Derek M.; Kahn, Jeremy M.; Olsen, Maren K.; Lewis, Carmen L.; Hanson, Laura C.; Carson, Shannon S.: Effects of a Personalized Web-Based Decision Aid for Surrogate Decision Makers of Patients With Prolonged Mechanical Ventilation: A Randomized Clinical Trial. Annals of internal medicine 2019
4. Curtis JR, Treece PD, Nielsen EL, Gold J, et al: Randomized Trial of Communication Facilitators to Reduce Family Distress and Intensity of End-of-Life Care. Am J Respir Crit Care Med 2016; 193(2):154-162
5. Kaufer MM, Patricia; Barker, Kris; Mosenthal, Anne: Family satisfaction following the death of a loved one in an inner city MICU. The American journal of hospice & palliative care 2008; 25(4):318-325
6. Kentish-Barnes N, Chevret S, Azoulay E: Guiding intensive care physicians' communication and behavior towards bereaved relatives: study protocol for a cluster randomized controlled trial (COSMIC-EOL). Trials 2018; 19(1):698
7. Lautrette AD, Michael; Megarbane, Bruno; Joly, Luc Marie; Chevret, Sylvie; Adrie, Christophe; Barnoud, Didier; Bleichner, Gerard; Bruel, Cedric; Choukroun, Gerald; Curtis, J. Randall; Fieux, Fabienne; Galliot, Richard; Garrouste-Orgeas, Maite; Georges, Hugues; Goldgran-Toledano, Dany; Jourdain, Merce; Loubert, Georges; Reignier, Jean; Saidi, Faycal; Souweine, Bertrand; Vincent, Francois; Barnes, Nancy Kentish; Pochard, Frederic; Schlemmer, Benoit; Azoulay, Elie: A communication strategy and brochure for relatives of patients dying in the ICU. The New England journal of medicine 2007; 356(5):469-478
8. Nielsen AHA, Sanne; Egerod, Ingrid; Lund, Trine Hojfeldt; Renberg, Marianne; Hansen, Torben Baek: The effect of family-authored diaries on posttraumatic stress disorder in intensive care unit patients and their relatives: A randomised controlled trial (DRIP-study). Australian critical care : official journal of the Confederation of Australian Critical Care Nurses 2019
9. Rosenbaum JLS, Joan R.; Yan, Yan; Abram, Nancy; Jeffe, Donna B.: Impact of a Neonatal-Bereavement-Support DVD on Parental Grief: A Randomized Controlled Trial. Death studies 2015; 39(1-5):191-200
10. Prigerson HG, Viola M, Brewin CR, Cox C, et al: Enhancing & Mobilizing the POtential for Wellness & Emotional Resilience (EMPOWER) among Surrogate Decision-Makers of ICU Patients: study protocol for a randomized controlled trial. Trials 2019; 20(1):408
11. White DB, Angus DC, Shields AM, Buddadhumaruk P, et al: A Randomized Trial of a Family-Support Intervention in Intensive Care Units. N Engl J Med 2018; 378(25):2365-2375

**Supplemental Table 4.** Guidelines for bereavement support from the UK National Institute for Health and Clinical Excellence^1^ and core outcomes set for evaluating bereavement support for adult caregivers in adult palliative care settings proposed by Harrop and colleagues^2^

| **Tiered Guidelines for Bereavement Support** | | | | |
| --- | --- | --- | --- | --- |
| *NICE Components* | *Level of Public Health Intervention* | *Type of Support* | *Support Provided By* | *Target Population and Level of Support Needed* |
| 1 | Universal | Information about bereavement and relevant supports | Family and friends (information supplied by health and social care professionals) | All bereaved (normal grief)  Low level of need |
| 2 | Selective or Targeted | Non-specialist support | Trained volunteers, mutual‐help groups, community supports | Those at‐risk of developing complex needs  Medium level of need |
| 3 | Indicated | Professional specialist interventions | Mental health services, bereavement services, or psychotherapy | Those with complex needs  High level of need |

| **Core Outcomes Set for Bereavement Support for Adult Caregivers in Adult Palliative Care Settings** | |
| --- | --- |
| *Ability to Cope with Grief* | |
| Negative and overwhelming grief | • Feelings of loneliness and emptiness  • Feelings of blame, guilt, anger, bitterness, regret  • Overwhelming thoughts and/or nightmares about loss  • Preoccupation with thoughts of the deceased |
| Communication and connectedness | • Ability to express feelings openly and honestly  • Feeling understood by and connected with other bereaved people |
| Understanding, accepting and finding meaning in grief | • Acceptance of grief experiences as normal  • Understanding, acceptance, finding meaning in loss  • Positive reminiscence and remembering of the deceased |
| Finding balance between grief and life going forwards | • Ability to find balance and channel grief  • Ability to take control/look ahead and start to move forward with life |
| Accessing appropriate support | • Accessing emotional support if needed  • Accessing practical support if needed |
| *Quality of Life and Mental Wellbeing* | |
| Participation in work and/or other regular activities | • Ability to perform daily tasks  • Ability to participate in work  • Ability to participate in social activities |
| Relationships and social functioning | • Ability to function as part of a family  • Relationships with friends and family |
| Positive mental wellbeing | • Sense of meaning and purpose in life  • Optimism and hopefulness |
| Negative mental and emotional state | • Anxiety (feelings of tension, nervousness, panic and distress)  • Depression (a sense of hopelessness, pessimism, periods of crying)  • Suicidal thoughts |

^1^Guidelines for Bereavement Support. Online, UK National Institute for Health and Clinical Excellence, 2020.

^2^Harrop E, Scott H, Sivell S, Seddon K, et al: Coping and wellbeing in bereavement: two core outcomes for evaluating bereavement support in palliative care. BMC Palliat Care 2020; 19(1):29

**Supplemental Table 5.** Results from Randomized Controlled Trials on the Association Between ICU Bereavement Interventions and Ability to Cope with Grief at 1- to 3-Month Follow-up

|  |  | **No. of Caregivers** | |  |  |  |
| --- | --- | --- | --- | --- | --- | --- |
| **Category** | **Author, Year** | **No. Intervention** | **No.**  **Control** | **Instrument** | **Standardized Mean Difference (95% CI)** | **Statistical Interpretation of Effect of Intervention** |
| *Negative and overwhelming grief* | | |  |  |  |  |
|  | Barnato et al., 2017 | 16 | 14 | Decision Regret Scale | -0.318 (-1.040 to 0.403) | No significant effect |
|  | Kentish-Barnes et al., 2017 | 109 | 99 | None |  |  |
|  | Tawil et al., 2014 | 38 | 20 | None |  |  |
| *Communication and connectedness* | | |  |  |  |  |
|  | Barnato et al., 2017 | 16 | 14 | None |  |  |
|  | Kentish-Barnes et al., 2017 | 109 | 99 | None |  |  |
|  | Tawil et al., 2014 | 38 | 20 | None |  |  |
| *Understanding, accepting and finding meaning in grief* | | | |  |  |  |
|  | Barnato et al., 2017 | 16 | 14 | None |  |  |
|  | Kentish-Barnes et al., 2017 | 109 | 99 | None |  |  |
|  | Tawil et al., 2014 | 38 | 20 | None |  |  |
| *Finding balance between grief and life going forwards* | | | |  |  |  |
|  | Barnato et al., 2017 | 16 | 14 | None |  |  |
|  | Kentish-Barnes et al., 2017 | 109 | 99 | None |  |  |
|  | Tawil et al., 2014 | 38 | 20 | None |  |  |
| *Accessing appropriate support* | | |  |  |  |  |
|  | Barnato et al., 2017 | 16 | 14 | Consulted with mental health specialist | -0.422 (-1.539 to 0.695) | No significant effect |
|  |  |  |  | Counseling for nerves, mood, sleep | -0.659 (-1.732 to 0.414) | No significant effect |
|  |  |  |  | Met with clergy | 0.033 (-0.9307 to 0.996) | No significant effect |
|  |  |  |  | Attended a grief support group | Not able to calcualate^1^ | Not able to determine |
|  | Kentish-Barnes et al., 2017 | 109 | 99 | None |  |  |
|  | Tawil et al., 2014 | 38 | 20 | None |  |  |

^1^No caregiver in the intervention or control group had attended a grief support group by 3-month follow up

**Supplemental Table 6.** Results from Randomized Controlled Trials on the Association Between ICU Bereavement Interventions and Ability to Cope with Grief at 4- to 6-Month Follow-up

|  |  | **No. of Caregivers** | |  |  |  |
| --- | --- | --- | --- | --- | --- | --- |
| **Category** | **Author, Year** | **No. Intervention** | **No.**  **Control** | **Instrument** | **Standardized Mean Difference (95% CI)** | **Statistical Interpretation of Effect of Intervention** |
| *Negative and overwhelming grief* | | |  |  |  |  |
|  | Barnato et al., 2017 | 17 | 13 | Decision Regret Scale | 0.230 (-0.494 to 0.955) | No significant effect |
|  | Kentish-Barnes et al., 2017 | 101 | 89 | None |  |  |
|  | Tawil et al., 2014 | Not applicable | Not applicable | Not applicable |  |  |
| *Communication and connectedness* | | |  |  |  |  |
|  | Barnato et al., 2017 | 17 | 13 | None |  |  |
|  | Kentish-Barnes et al., 2017 | 101 | 89 | None |  |  |
|  | Tawil et al., 2014 | Not applicable | Not applicable | Not applicable |  |  |
| *Understanding, accepting and finding meaning in grief* | | | |  |  |  |
|  | Barnato et al., 2017 | 17 | 13 | Inventory of Complicated Grief | 0.045 (-0.698 to 0.747) | No significant effect |
|  | Kentish-Barnes et al., 2017 | 101 | 89 | Inventory of Complicated Grief | 0.868 (0.569 to 1.166) | Significantly worsened |
|  | Tawil et al., 2014 | Not applicable | Not applicable | Not applicable |  |  |
| *Finding balance between grief and life going forwards* | | | |  |  |  |
|  | Barnato et al., 2017 | 17 | 13 | None |  |  |
|  | Kentish-Barnes et al., 2017 | 101 | 89 | None |  |  |
|  | Tawil et al., 2014 | Not applicable | Not applicable | Not applicable |  |  |
| *Accessing appropriate support* | | |  |  |  |  |
|  | Barnato et al., 2017 | 17 | 13 | Consulted with mental health specialist | -0.659 (-1.726 to 0.408) | No significant effect |
|  |  |  |  | Counseling for nerves, mood, sleep | -0.422 (-1.537 to 0.693) | No significant effect |
|  |  |  |  | Met with clergy | -0.422 (-1.537 to 0.693) | No significant effect |
|  |  |  |  | Attended a grief support group | -0.091 (-1.703 to 1.421) | No significant effect |
|  | Kentish-Barnes et al., 2017 | 101 | 89 | None |  |  |
|  | Tawil et al., 2014 | Not applicable | Not applicable | Not applicable |  |  |

**Supplemental Table 7.** Results from Randomized Controlled Trials on the Associations Between ICU Bereavement Interventions and Quality of Life and Mental Wellbeing at 1- to 3-Month Follow-up

|  |  | **No. of Caregivers** | |  |  |  |
| --- | --- | --- | --- | --- | --- | --- |
| **Category** | **Author, Year** | **No. Intervention** | **No.**  **Control** | **Instrument** | **Standardized Mean Difference (95% CI)** | **Statistical Interpretation of Effect of Intervention** |
| *Participation in work and/or other regular activities* | | |  |  |  |  |
|  | Barnato et al., 2017 | 16 | 14 | None |  |  |
|  | Kentish-Barnes et al., 2017 | 109 | 99 | None |  |  |
|  | Tawil et al., 2014 | 38 | 20 | General Health Questionnaire-12 | -0.065 (-0.606 to 0.477) | No significant effect |
| *Relationships and social functioning* | | |  |  |  |  |
|  | Barnato et al., 2017 | 16 | 14 | None |  |  |
|  | Kentish-Barnes et al., 2017 | 109 | 99 | None |  |  |
|  | Tawil et al., 2014 | 38 | 20 | None |  |  |
| *Positive mental wellbeing* | | | |  |  |  |
|  | Barnato et al., 2017 | 16 | 14 | None |  |  |
|  | Kentish-Barnes et al., 2017 | 109 | 99 | None |  |  |
|  | Tawil et al., 2014 | 38 | 20 | None |  |  |
| *Negative mental and emotional state* | | |  |  |  |  |
|  | Barnato et al., 2017 | 16 | 14 | Impact of Event Scale - Revised | 0.011 (-0.707 to 0.728) | No significant effect |
|  |  |  |  | Hospital Anxiety and Depression - Anxiety | -0.201 (-0.920 to 0.518) | No significant effect |
|  |  |  |  | Hospital Anxiety and Depression - Depression | 0.148 (-0.569 to 0.866) | No significant effect |
|  |  |  |  | Patient Health Questionnaire-9^1^ | 0.552 (-0.789 to 1.893) | No significant effect |
|  |  |  |  | Post-Intensive Care Syndrome-Family^2^ | -0.024 (-0.845 to 0.798) | No significant effect |
|  | Kentish-Barnes et al., 2017 | 109 | 99 | Impact of Event Scale - Revised | Not assessed | Not assessed |
|  |  |  |  | Hospital Anxiety and Depression - Depression | 0.999 (0.711 to 1.288) | No significant effect |
|  |  |  |  | Hospital Anxiety and Depression - Total | 0.841 (0.557 to 1.125) | No significant effect |
|  | Tawil et al., 2014 | 38 | 20 | Impact of Event Scale | 0.229 (-0.314 to 0.772) | No significant effect |

^1^Proportion of caregivers with scores suggestive of severe depression (i.e., ≥20)

^2^Scores equal to or greater than scores proposed as cut-offs suggestive of syndromic burden on Hospital Anxiety and Depression – Anxiety ≥8; Hospital Anxiety and Depression - Depression ≥8; Impact of Event Scale – Revised ≥24; and/or Inventory of Complicated Grief ≥25

**Supplemental Table 8.** Results from Randomized Controlled Trials on the Associations Between ICU Bereavement Interventions and Quality of Life and Mental Wellbeing at 4- to 6-Month Follow-up

|  |  | **No. of Caregivers** | |  |  |  |
| --- | --- | --- | --- | --- | --- | --- |
| **Category** | **Author, Year** | **No. Intervention** | **No.**  **Control** | **Instrument** | **Standardized Mean Difference (95% CI)** | **Statistical Interpretation of Effect of Intervention** |
| *Participation in work and/or other regular activities* | | |  |  |  |  |
|  | Barnato et al., 2017 | 17 | 13 | None |  |  |
|  | Kentish-Barnes et al., 2017 | 101 | 89 | None |  |  |
|  | Tawil et al., 2014 | Not applicable | Not applicable | Not applicable |  |  |
| *Relationships and social functioning* | | |  |  |  |  |
|  | Barnato et al., 2017 | 17 | 13 | None |  |  |
|  | Kentish-Barnes et al., 2017 | 101 | 89 | None |  |  |
|  | Tawil et al., 2014 | Not applicable | Not applicable | Not applicable |  |  |
| *Positive mental wellbeing* | | | |  |  |  |
|  | Barnato et al., 2017 | 17 | 13 | None |  |  |
|  | Kentish-Barnes et al., 2017 | 101 | 89 | None |  |  |
|  | Tawil et al., 2014 | Not applicable | Not applicable | None |  |  |
| *Negative mental and emotional state* | | |  |  |  |  |
|  | Barnato et al., 2017 | 17 | 13 | Impact of Event Scale - Revised | -0.044 (-0.766 to 0.679) | No significant effect |
|  |  |  |  | Hospital Anxiety and Depression - Anxiety | -0.194 (-0.918 to 0.530) | No significant effect |
|  |  |  |  | Hospital Anxiety and Depression - Depression | -0.018 (-0.739 to 0.705) | No significant effect |
|  |  |  |  | Patient Health Questionnaire-9^1^ | Not able to calculate | Not able to determine |
|  |  |  |  | Post-Intensive Care Syndrome-Family^2^ | -0.375 (-1.209 to 0.459) | No significant effect |
|  | Kentish-Barnes et al., 2017 | 101 | 89 | Impact of Event Scale - Revised | 1.323 (1.009 to 1.638) | Significantly worsened |
|  |  |  |  | Hospital Anxiety and Depression - Depression | 1.789 (1.452 to 2.126) | Significantly worsened |
|  |  |  |  | Hospital Anxiety and Depression - Total Scale | 1.380 (1.063 to 1.697) | Significantly worsened |
|  | Tawil et al., 2014 | Not applicable | Not applicable | Not applicable |  |  |

^1^Proportion of caregivers with scores suggestive of severe depression (i.e., ≥20); *n*=0 (0%) caregivers have a PHQ-9 score ≥20 at 6-month follow-up

^2^Scores equal to or greater than scores proposed as cut-offs suggestive of syndromic burden on Hospital Anxiety and Depression – Anxiety ≥8; Hospital Anxiety and Depression - Depression ≥8; Impact of Event Scale – Revised ≥24; and/or Inventory of Complicated Grief ≥25
